# Paralytic Shellfish Toxin Uptake, Assimilation, Depuration, and Transformation in the Southeast Asian Green-Lipped Mussel (*Perna viridis*)

**DOI:** 10.3390/toxins11080468

**Published:** 2019-08-09

**Authors:** John Kristoffer Andres, Aletta T. Yñiguez, Jennifer Mary Maister, Andrew D. Turner, Dave Eldon B. Olano, Jenelyn Mendoza, Lilibeth Salvador-Reyes, Rhodora V. Azanza

**Affiliations:** 1 The Marine Science Institute, University of the Philippines Diliman, Quezon City 1101, Philippines; 2 Food Safety Group, Centre for Environment, Fisheries and Aquaculture Science, Barrack Road, Weymouth, Dorset DT4 8UB, UK

**Keywords:** saxitoxin, harmful algal blooms, biotransformation, uptake, depuration, assimilation, shellfish, *Perna viridis*, *Alexandrium*

## Abstract

Bivalve molluscs represent an important food source within the Philippines, but the health of seafood consumers is compromised through the accumulation of harmful algal toxins in edible shellfish tissues. In order to assess the dynamics of toxin risk in shellfish, this study investigated the uptake, depuration, assimilation, and analogue changes of paralytic shellfish toxins in *Perna viridis*. Tank experiments were conducted where mussels were fed with the toxic dinoflagellate *Alexandrium minutum*. Water and shellfish were sampled over a six day period to determine toxin concentrations in the shellfish meat and water, as well as algal cell densities. The maximum summed toxin concentration determined was 367 µg STX eq./100 g shellfish tissue, more than six times higher than the regulatory action limit in the Philippines. Several uptake and depuration cycles were observed during the study, with the first observed within the first 24 h coinciding with high algal cell densities. Toxin burdens were assessed within different parts of the shellfish tissue, with the highest levels quantified in the mantle during the first 18 h period but shifting towards the gut thereafter. A comparison of toxin profile data evidenced the conversion of GTX1,4 in the source algae to the less potent GTX2,3 in the shellfish tissue. Overall, the study illustrated the temporal variability in *Perna viridis* toxin concentrations during a modelled algal bloom event, and the accumulation of toxin from the water even after toxic algae were removed.

## 1. Introduction

Paralytic shellfish poisoning (PSP) is caused by the consumption of shellfish such as bivalve molluscs contaminated with paralytic shellfish toxins (PST), a family of compounds related to saxitoxin (STX) which are produced naturally by several species of dinoflagellates. Uptake and depuration of toxins within the flesh of the molluscs varies greatly from one shellfish species to another [1,2,3,4,5], with toxin retention lasting from days to months, depending on the species [5]. Differences in the accumulation of PST between different bivalve species has been reported during a bloom of *Pyrodinium bahamense* var. *compressum* (PBC) in Masinloc Bay, Philippines [6]. Seven species of bivalves were tested for toxicity where six of them became toxic. The species *Spondylus squamosus* obtained the highest toxicity during the peak of the bloom and several cycles of uptake and depuration of toxin were observed within a year. *S. squamosus* was also able to uptake toxins even in the absence of the causative phytoplankton, with toxicity eventually increasing following another algal bloom. The same behaviour was observed in *Atrina vexellum* but at lower magnitudes. As for the green-lipped mussel, *Perna viridis*, the pattern for toxicity seemed to follow the cycle for PBC wherein during the onset of the bloom, toxicity was seen to increase in the shellfish tissue, then decreasing when the bloom of PBC declined.

Toxins are taken inside the shellfish system through filter-feeding activity but are not distributed evenly across different parts of the tissue [2]. Depending on the time of toxin exposure, the burden or amount of overall toxicity per part shifts from one organ to another. Generally, the viscera (organs in the abdominal cavity including the digestive gland), show the highest toxicity [1,2,7]. A five-compartment model has been developed for *Perna viridis* fed with *Alexandrium tamarense* [7]. The mussel was dissected into five parts, namely the hepatopancreas, viscera, gill, adductor muscle, and foot. The highest toxin levels were determined in the hepatopancreas (47–74% of toxicity), followed by the viscera (8–41%), gill (2–18%), adductor muscle (1–13%), and foot (0.4–5%). Interestingly, the organ group with the lowest contribution to the total shellfish body mass had the highest level of toxicity. The high relative concentrations of toxins in the hepatopancreas relate to the fact that this organ is responsible for toxin removal from the shellfish system. The distribution of PST in *Perna viridis* and the scallops *Chlamys nobilis* fed with *Alexandrium tamarense* has also been assessed [8]. The mussel was dissected into the digestive gland and other parts while the scallop was dissected into adductor muscle and other tissues. Results highlighted that in mussels, the digestive gland contained the highest toxin burden. As for the scallops, toxin burden was higher in the other tissues compared to the adductor muscle. Two phases of depuration were observed for both shellfish species. The first phase was characterised by fast depuration, which was presented as the gut evacuation of the unassimilated toxins. The second phase was slower, which was thought related to be depurating toxins becoming assimilated and incorporated into other tissues.

Transformation of saxitoxin analogues has been observed by monitoring the differences in toxin profiles detected in both the source phytoplankton and shellfish tissue [2]. Biotransformation is thought to usually occur during periods of detoxification or contamination [9]. Changes in toxin profiles within shellfish tissues may occur either through selective retention of specific toxin analogues, or through enzymatic transformation, indicating active toxin metabolism in shellfish [10]. PSTs undergo transformation from one form to another through different processes. Such processes include reduction, epimerisation, oxidation, and, desulfation, all of which may potentially result in changes to overall shellfish toxicity [3], following the conversion of toxins to other analogues of lower or higher potency. Conversion to less potent forms may accelerate detoxification through the flushing out of toxin from the shellfish system, while conversion to more potent forms will result in a slower detoxification rate [9]. Many studies have shown the ability of *Perna viridis* as well as other species such as *Chlamys nobilis*, to convert STX analogues to more potent forms [11]. Epimerisation has also been detected through the conversion of predominantly C2 in *A. tamarense* to C1 in both shellfish species [8]. In addition, new metabolites were found in the shellfish that were not present in the source phytoplankton.

Different methods are used to detect and quantify PST, including animal bioassays, chemical detection methods and immunoassays [12]. The mouse bioassay (MBA) has for many decades been the reference method used for toxin monitoring programmes in most countries and for many years has been the official control reference method in both the EU and the US. Chemical detection methods such as high-performance liquid chromatography with fluorescence detection (HPLC-FLD) enable the quantitation of individual toxins or epimeric pairs, facilitating the calculation of total sample toxicity using appropriate toxicity equivalence factors (TEFs). To date two separate HPLC-FLD methods have been developed and validated through collaborative study, both using sample derivatization. The first, post-column oxidation HPLC-FLD was developed in Canada and has been accepted for official control testing by the ISSC for use in the US and Canada [13]. The second, known as the Lawrence method, utilizes pre-column oxidation with HPLC-FLD and starting 1st January 2019 became the official reference method for PSP analysis within the EU [14]. More recently mass spectrometry-based methods have been developed and validated [15] enabling the rapid quantitation of a wider range of analogues.

HABs occur globally throughout all the major continents, with the South China Sea known to be one of the world’s hotspots for HAB occurrences [16]. However, there are limited studies in the region involving the dynamics and kinetics of HAB toxins, particularly PST in bivalves. The major toxin producers for this region are *Alexandrium sp.* including species originally assigned as *Gonyaulux sp.* [8]. These species produce primarily N-sulfocarbamoyl PST and C toxins, with C2 as the dominant toxin type. There is minimal information, however, on the toxin dynamics of *Perna viridis* as it interacts with toxic *Alexandrium* species. Since *P. viridis* is one of the main contributors to aquaculture production in the Philippines, and also serves as a cheap source of food for the local population, it is important to determine the risk of toxicity from this species both from a health and socio-economic perspective. Consequently, this study sought to assess the pattern of uptake and depuration of toxins within the mussels, the distribution patterns of toxins within the shellfish tissue and the presence and implications of toxin transformation. These results, obtained through laboratory feeding experiments, would then be used to assess the overall risks from PST in mussels from the Philippines.

## 2. Results

### 2.1. Overall Toxicity, Uptake, and Depuration

**Figure 1:**
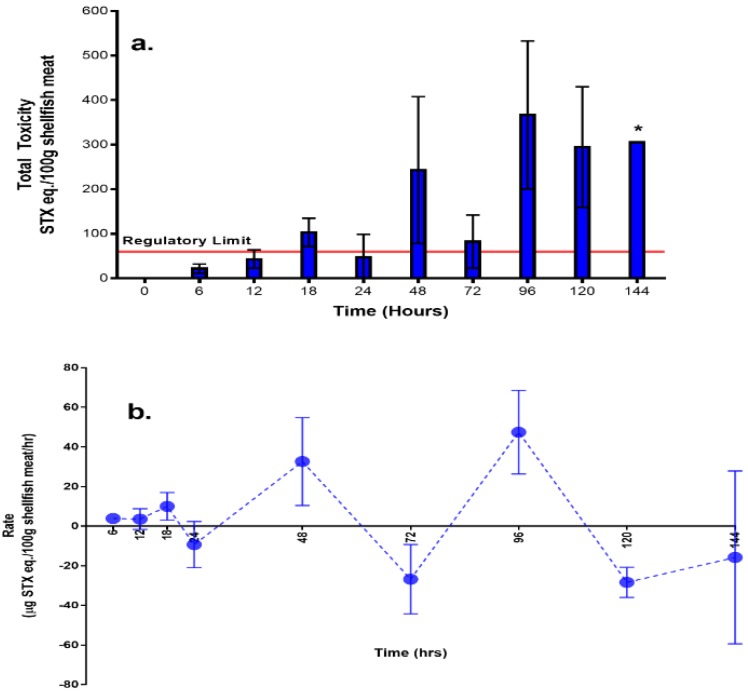
Paralytic shellfish toxins (PST) quantity and patterns of uptake and depuration for the shellfish in tank A (phytoplankton + shellfish set-up) (**a**) total toxicity; (**b**) rate. (* no standard error since only one tank was sampled due to shellfish mortality).

The total PST concentrations were used to estimate total toxicity in each of the shellfish samples analysed. Mussels sampled from the negative control tanks (Tank C containing shellfish only) were found to contain low levels of GTX1,4; and GTX2,3 analogues due to their prior exposure in the field. Therefore, the average toxicity values obtained from the negative control tanks were subtracted from the values of toxicity from the phytoplankton + shellfish set-up for each sampling period to give an accurate estimation of toxin uptake. The regulatory maximum permitted limit (MPL) for PST in the Philippines is 60 µg saxitoxin equivalents per 100 g shellfish meat (600 µg STX eq./kg). Total shellfish toxin concentrations determined here were generally lower than the MPL within the first six hours of the feeding study. From the 12th hour sampling point until the end of the experiment, total PST exceeded the MPL with the highest total toxin levels occurring in mussels sampled after 96 h, containing 367 (±166.17) µg STX eq./100 g (Figure 1a and Supplementary Material Table S1).

The uptake and depuration rates for total PST in mussels following exposure to toxic *A. minutum* cycled three times (Figure 1b). The first cycle of uptake and depuration occurred within the first 24 h of the experiment. The second and third cycles of uptake were then repeated during the 48th hour and 96th hour of sampling, respectively, while depuration occurred at the 72nd and 120th hour. Interestingly, the uptake rates before depuration occurred had similar values (from 8–11 µg STX eq./h), implying a threshold uptake rate for shellfish.

**Figure 2:**
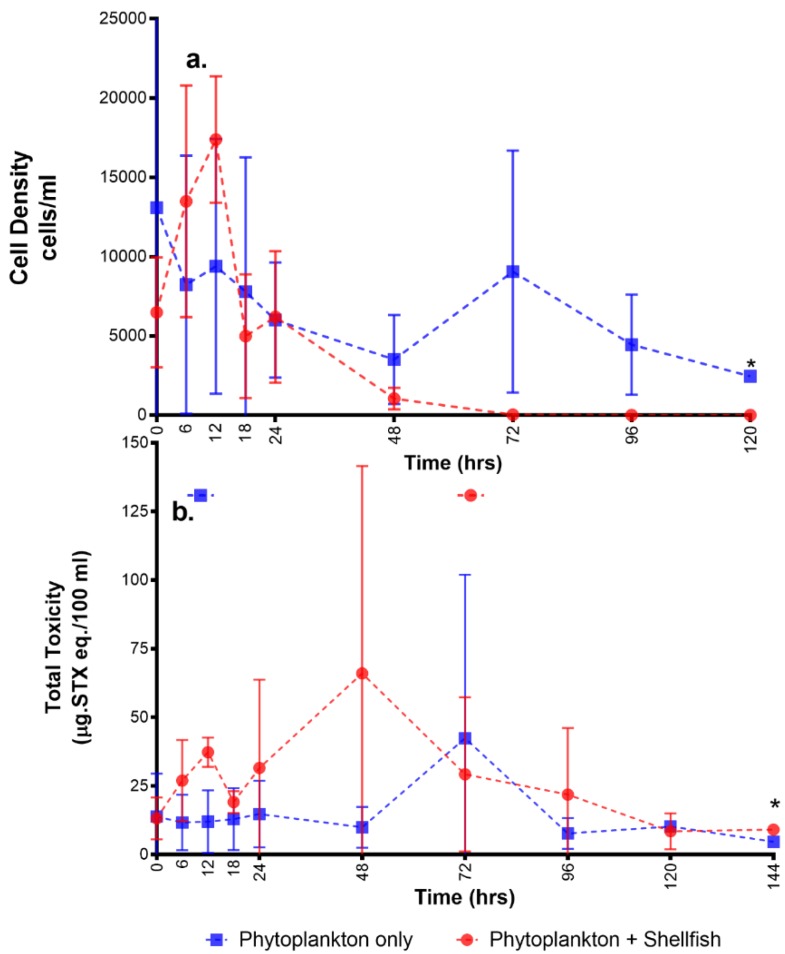
Parameters measured in the water: (**a**) cell density; (**b**) total toxicity. (* no standard error since only one tank was sampled due to shellfish mortality).

The mussels within the experimental tanks can potentially accumulate toxins from two sources: (1) the *A. minutum* cells; and (2) any toxins dissolved in the water. The *A. minutum* cell densities in tank A initially increased but began to decrease at the 12th hour. By the 72nd hour onwards, there were no cells present since the water in the tanks was changed to fresh seawater, free from toxic algae (Figure 2a). The cell densities in tank B, the phytoplankton control, showed a similar trend at the start. However, the cells remained beyond the 72nd hour since water here was not changed. In the water column of the tanks, overall toxicity exhibited some increases and decreases (Figure 2b and Supplementary Material Table S2). Tank B showed a peak in toxin levels during the 72nd hour, while for the phytoplankton + shellfish tank, a peak occurred at the 48th hour and a smaller peak was also observed during the 12th hour. Variability in the toxin concentrations was high and no significant difference was observed in the overall toxin concentrations in water samples between the phytoplankton only tanks (tank B) and those with phytoplankton + shellfish (tank A). The average toxin value recorded for water in tank B was 11.1 µg STX eq./100 mL (range: 1–26 µg STX eq./100 mL), while for the water in tank A, the average toxicity was higher at 23.5 µg STX eq./100 mL (range: 3–67 µg STX eq./100 mL).

### 2.2. Toxin Compartmentalisation

**Figure 3:**
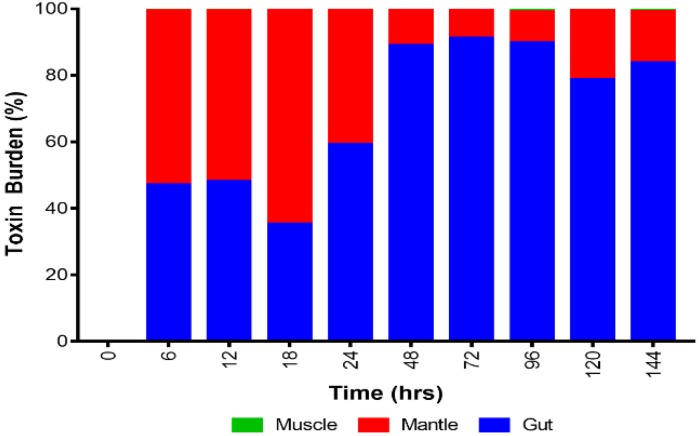
The distribution of toxin at different parts of the shellfish through time.

The toxin distributions quantified within the shellfish tissues varied temporally and between parts (Figure 3 and Supplementary Material Figure S1). Six hours after feeding, the mantle and the gut contained similar toxin burdens at 52% and 47%, respectively. The part of the mussels containing the lowest toxin concentrations was the foot and adductor muscle (0.7%). The same toxin burden pattern was seen from the 12th hour to the 18th hour. From the 24th hour of the experiment to the 144th hour, toxin burden shifted more towards the gut (59–91%), followed by the mantle (8–40%), and again, the least burden was determined in the muscle (0.12–1.05%). Significant differences in the overall toxicity values between the gut and muscle and mantle and muscle were observed (Kruskal–Wallis, *p*-value < 0.05). No significant difference in the toxin concentration was found between gut and mantle

### 2.3. Toxin Types and Their Distributions

**Figure 4:**
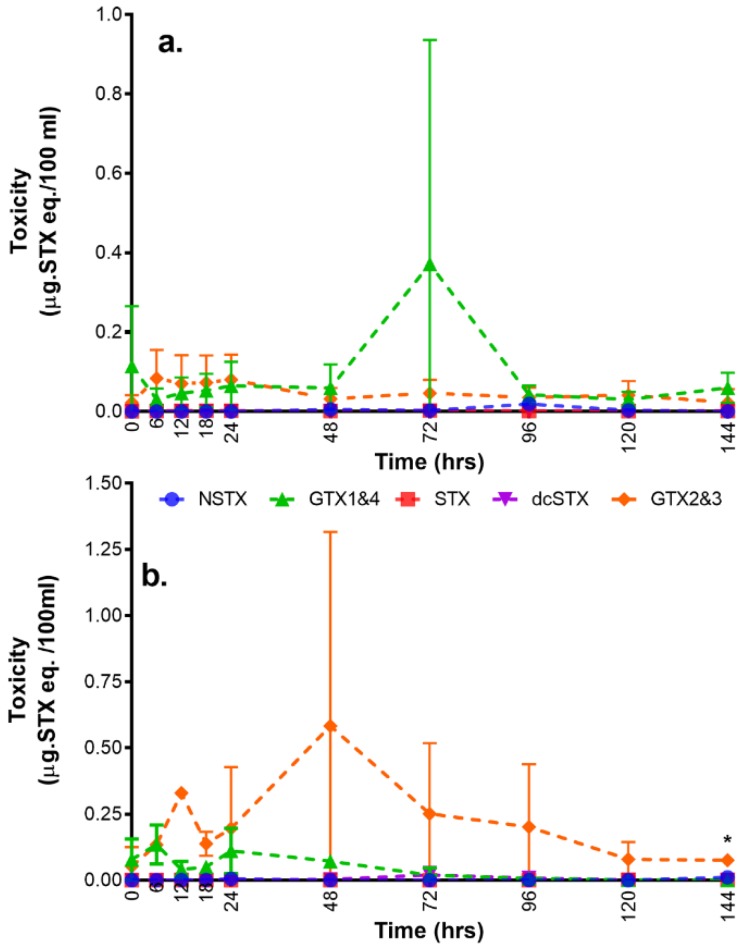
The patterns of saxitoxin analogues in water through time at (**a**) phytoplankton only set-up and (**b**) phytoplankton + shellfish. (* no standard error since only one tank was sampled due to shellfish mortality).

The toxin profile of *A. minutum* from the phytoplankton only set-up was dominated by GTX1,4; with traces of GTX2,3 (Figure 4 and Supplementary Material Table S3). In the tanks containing phytoplankton and shellfish, the toxin profiles were reversed with GTX2,3 dominating; and with lower relative proportions of GTX1,4.

The PST analogue concentrations determined in the shellfish meat are summarised in Table 1. Representative chromatograms can be found in Figure S2 of the Supplementary Material GTX1,4; and GTX2,3 showed the highest toxin concentration values, whilst other analogues were present at either low concentrations or were non-detectable. Thus, GTX1,4; and GTX2,3 were used to more closely investigate the toxicity patterns in the shellfish meat.

**Figure 5:**
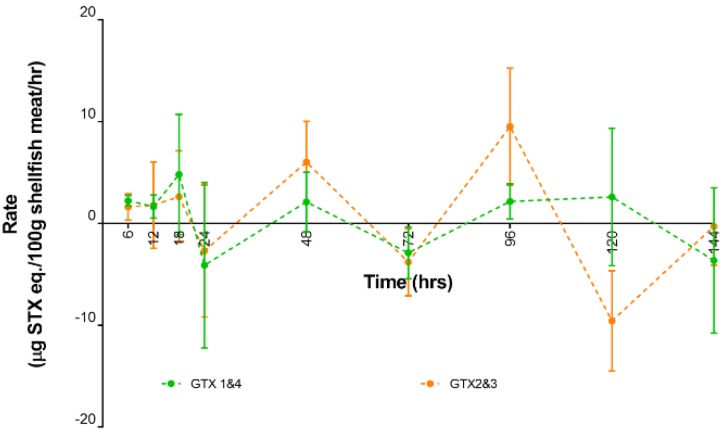
Uptake and depuration rate for GTX1,4; and GTX2,3.

Looking more closely at the uptake and depuration rates for GTX1,4; and GTX2,3; three uptake cycles were also apparent (Figure 5). From the start of the experiment until the 96th hour, uptake and depuration for both toxins occurred synchronously. However, during the 120th hour, a further increase in GTX1,4 was observed whilst conversely there was a depuration loss of GTX2,3. A reverse in uptake and depuration between two GTX analogues was also observed at the 144th hour when uptake was observed for GTX2,3; and depuration occurred for GTX1,4.

**Figure 6:**
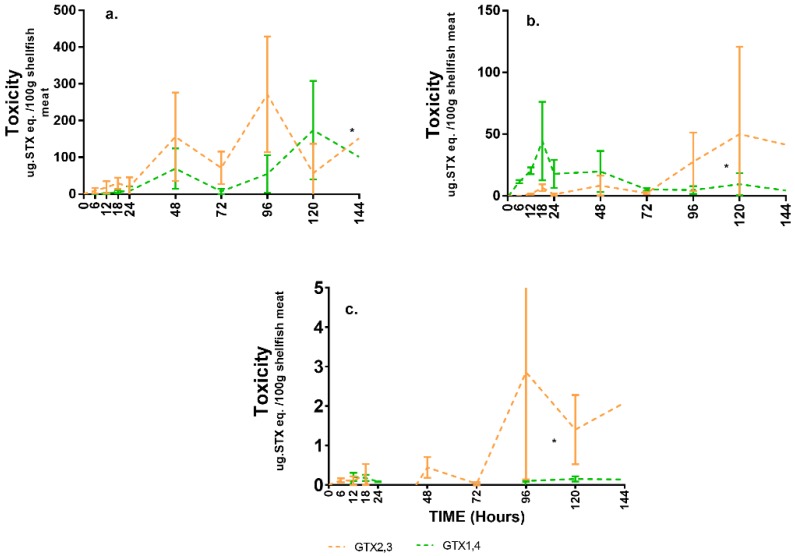
Concentration of GTX1,4 and GTX2,3 through time at (**a**) gut; (**b**) mantle; and (**c**) muscle. (*no standard error since only one tank was sampled due to shellfish mortality).

The gut and muscle showed the same toxin analogue profile where GTX2,3 tended to have higher concentrations relative to GTX1,4. The highest average toxicity recorded for the gut was 271 µg STX eq./100 g shellfish meat, while the muscle had a much lower value of 2.8 µg STX eq./100 g. In the gut, GTX2,3 was relatively higher already at the 48th hour onwards, while toxicity in the muscle became more pronounced during the latter part of the study (Figure 6a,c). For the mantle, GTX1,4 had the highest concentration at the start of the experiment from 0–48 h. Toxin analogues shifted towards GTX2,3 from 72nd hour onwards (Figure 6b).

Qualitatively the difference in terms of overall water toxicity and PST analogues can be seen, though statistically there were no significant differences (average total toxicity and saxitoxin analogues between times; multiple *t*-test). This is likely due to the high variability between shellfish samples and/or the lower power of non-parametric statistics. This high inter-individual variability among shellfish has been observed frequently [3,7,8,17,18,19] and one potential explanation is the diverse physiological processes leading to sensitivity of shellfish to PST toxins [17].

## 3. Discussion

### 3.1. Uptake and Depuration Patterns

In a period less than 24 h, toxin uptake began, suggesting that this species can respond rapidly to the presence of the toxic phytoplankton. Bivalve feeding rhythms are thought to be based on food availability, with previous work demonstrating that there are cycles of clearance rate which are seen to occur within 24 h [11]. This cyclical pattern of uptake and depuration may represent the natural feeding behaviour of shellfish. Within the first 6 h of the feeding study, toxicity was generally lower than the national regulatory MPL. However, subsequent measurements starting from the 12th hour showed total mussel toxicity to rise above this limit. Based on the cycles of uptake and depuration, *P. viridis* is capable of depurating within a period of 24 hours. Moreover, repeated cycles of uptake and depuration were observed up to the 144th hour, the last sampling period. Due to the limitations in experimental design, the time to which the toxin will return below the MPL cannot be determined. From previous reports, however, toxin accumulation and elimination are relatively rapid in mussels, taking from days to weeks. In scallops and butter clams, elimination is known to be slower, sometimes over one year, due to toxin binding within the siphon tissue of animals [2,3,7]. One of the few studies that looked at short-term responses examined the uptake of Azaspiracids (AZAs) in scallops and mussels. The initial uptake was recorded from 24–48 h of the study, followed by depuration between 48–72 h. The cycle of uptake and depuration was recorded again during the next sampling periods with the same interval [20]. Moreover, the cycles for uptake and depuration show a similar pattern to the levels of total toxicity recorded. Interestingly, the results of our study showed that there appears to be a threshold value for the uptake rate (8–11 µg STX eq./hr) before depuration proceeds. This pattern can also be the result of the one-time feeding in the experiment, and could also represent conditions in the field wherein there is a pulse of high phytoplankton concentration which then declines as the bloom dies off. The highly variable toxin concentrations can pose a problem for HAB toxin monitoring programmes since the measured toxin would be dependent on the timing relative to the uptake and depuration cycle of the mussels. In addition, water containing toxic cells was removed on the 72nd hour in the tank A set-ups and were replaced with toxin-free UV-sterilized seawater. However, the shellfish were still able to accumulate toxins even after the removal of toxic cell source. Since the set-up was a closed-system, these toxins may have come from the dead mussels or depuration from other mussels, which could then have been taken up again by the remaining *P. viridis*.

### 3.2. Toxin Distribution within Shellfish

The toxins quantified in the shellfish taken from the toxic shellfish tank system were not equally distributed. This is important to determine given that the mussels are harvested and separated into specific parts prior to consumption [5]. For the first 18 h of the study, toxin burden in the mantle was the highest with 51–64% of the total toxin content. This was followed by the gut and lastly by the muscle group with 35–47% and 0.51–0.78% toxicity respectively. Changes in toxin profiles were observed after 24 h wherein the gut contained the highest toxin levels (59–91%). The mantle had 8–40% while the muscle group had 0.12–1.05% toxin burden recorded up until the last sampling period. The patterns observed here conform to the general observation that PSP toxins tend to accumulate the highest in the viscera [1,3,4,5] and that the distribution of toxins among tissues may shift depending on the time of exposure to toxins [4]. Despite the low contribution of viscera to the total soft tissue mass, it still has the highest toxicity. The viscera is the first organ exposed to the toxic cells and where toxins are absorbed, ingested, and metabolized [5]. In contrast, the foot and adductor muscle contribute to the majority of the soft tissue mass of the shellfish but was found to have the lowest toxicity. Depending on the time of exposure to toxic phytoplankton, the toxin levels present in each part of the molluscs changed, resulting in changes to toxin profiles between compartments. In other previous studies describing the toxification phases of *S. giganteus, M. mercenaria, S. solidissima, P. magellanicus*, and *Mya arenaria*, the viscera was reported to contain the highest toxin burden and shifted towards the siphon-gill tissue during detoxification phases. In *Mytilus edulis*, the viscera contained the highest toxin concentrations throughout the process [5]. The results of this study therefore conformed to the general observations that the gut had the highest toxin burden [1,3,4,5,8]. This may be attributed to the role of the gut to breakdown toxins from toxic algae. These observations therefore highlight the importance of appropriate mussel preparation when processing contaminated mussels for human consumption.

### 3.3. Toxin Transformation

Certain specific bivalve species are able to biotransform PST analogues following uptake of toxins from phytoplankton during filter-feeding, resulting in the generation of metabolites not found within the source algae [2,5,10,21,22]. Due to practical limitations in relation to access to commercial reference standards, only five saxitoxin analogues were assessed during this study. These analogues are considered to be the most potent PSTs. The dominant analogue detected in the water of the tanks containing only *A. minutum* was GTX1,4 with only traces of GTX2,3. Algal profiles were therefore similar to those of some previous studies [23], although toxin profiles in *A. minutum* are known to vary significantly throughout the world falling into a number of distinct clusters [24]. Conversely, however, GTX2,3 was found to be higher in the water sampled from the tanks containing shellfish and phytoplankton, as well as in the tissue from the shellfish in the same tanks. This suggests that biotransformation had occurred to convert GTX1,4 to GTX2,3. Analysis of samples was performed during the same analytical batch demonstrating these variations were not related to any aspect of method repeatability. Similarly, a shift in the toxin analogues detected in *Perna viridis* has been previously reported following feeding with *A. fundyense*. The source algae had a high ratio of GTX2/GTX3, but this ratio decreased in the shellfish samples indicating active epimerization within the shellfish tissue, prior to later transformation to STX through reduction [25]. Complex mechanisms involved in selective accumulation and chemical/enzymatic processes may be involved in the shellfish toxicity development [26]. These changes in analogues usually occur during periods of detoxification or contamination [9]. Different processes for toxin biotransformation include reduction, epimerization, oxidation, and desulfation [2,3,4,5,21,22]. The biotransformation potentially observed in this study resulted in the conversion to a less potent (GTX 2,3) from a more potent (GTX1,4) form. Transformation from GTX1,4 to GTX2,3 may be due to the epimerization process [27], and/or with the help of enzymes [28]. In addition, transformation is reported through reductive elimination with the decrease of N–OH group and increase in N–H group and elimination of sulfate group at the C11 position [29]. These transformations could be attributed to the shellfish itself or possibly bacterial action inside the shellfish [17] with the exact mechanisms in *P. viridis* requiring further investigation. Ideally any future work should incorporate a full suite of analytical standards into the detection method, with LC-MS/MS being utilised for a better understanding of the biotransformation processes within the shellfish tissues. Overall, the toxin profile data quantified in algae and mussels shows good evidence for transformation of toxin analogues, indicating the potential for species-specific transformation reactions similar to a number of other bivalve mollusc species.

From a more speculative ecological perspective, areas with high seawater residence time including many HAB-affected embayments in the Philippines, may encounter conditions where the toxin remains in the area due to the poor flushing of the water. Prolonged shellfish bans and toxicity in the shellfish have been observed in the Philippines even when the blooms of toxic cells have disappeared. These could be due to the ingestion of cysts [18,30] or illustrated here, through toxins that are present in the water column. This suggests that in our HAB monitoring programmes it will be advisable not only to measure HAB species cell densities, but also, at least initially, the toxin concentrations present in the water using technologies such as SPATT [31]. In addition, temporal and within-shellfish variabilities in toxin concentration can be further considered in designing HAB toxin monitoring programmes since the toxin levels measured could significantly change depending on the geographical and temporal nature of the sampling conducted.

## 4. Conclusions

Harmful algal blooms can plague certain areas in the Philippines for extended periods of time, resulting in a number of shellfish species accumulating toxins that present significant health and socio-economic risks. Here we show that in less than 24 h, *P. viridis* was able to uptake toxins when fed with toxic algae and consistently remained above the regulatory MPL in the Philippines from the 12th hour of the study onwards. Uptake and depuration rates cycled several times, which could represent a threshold value for the rate when shellfish starts to depurate and/or be related to the inconsistent supply of toxic *A. minutum* cells used for feeding. Even though toxic cells were removed, an increase in toxin concentrations in the shellfish were observed, which likely came from the toxin available in the water. This toxin could originate from dead mussels, faeces, and/or the depuration of toxin from the remaining toxin-containing mussels. In terms of the toxin distribution throughout the mussel tissue compartments, toxin profiles varied depending on the time of exposure to toxic cells. Initially, the mantle had the highest toxin burden, but later shifted towards the gut after 24 h. The toxin profiles of the source phytoplankton were found to be different from the profiles determined in both the shellfish tissue itself and the water sampled from the tanks containing shellfish fed with phytoplankton. GTX1,4 were dominant in the source algae with traces of GTX2,3, while the reverse was observed in both shellfish meat and water of the shellfish fed with phytoplankton setup. This indicates that biotransformation potentially occurred leading to a less toxic form.

Overall this study confirms the importance of both a water and shellfish monitoring programme for managing the risks from paralytic shellfish toxins in mussels from the Philippines. The current sampling design in toxin monitoring programmes of HAB affected areas should likely be re-assessed in order to help ensure food safety. Another key consideration is to test levels of toxins dissolved in seawater in addition to cell counts and shellfish toxicity. This may help shed light on observations of prolonged shellfish bans even if toxic cells were no longer detected. Similar studies are also needed for other shellfish species commonly consumed in the country.

## 5. Materials and Methods

### 5.1. Cell Cultures

Cultures of *Alexandrium minutum* were obtained from standing stock cultures (A.minBat). Cells were then sub-cultured to bigger flasks every three weeks until the volume reached six 80 L of culture of *A. minutum*. Culturing of cells to reach the final volume took approximately 18 weeks to be completed.

### 5.2. Acclimation

*Perna viridis* mussels were obtained from Bolinao, Pangasinan where HAB events occur regularly. Mussels were acclimatised for four days in a seawater aquarium with aerators, with temperature and salinity regulated to 27 °C and 32 ppt, respectively, whilst being fed daily with cultures of non-toxin producing *Isochrysis galbana*. Water was replaced every day to prevent accumulation of mucus that can cause mussel death. After 4 days, mussels were starved for 24 h prior to the grazing experiment commencing.

### 5.3. Grazing Experiment

**Figure 7:**
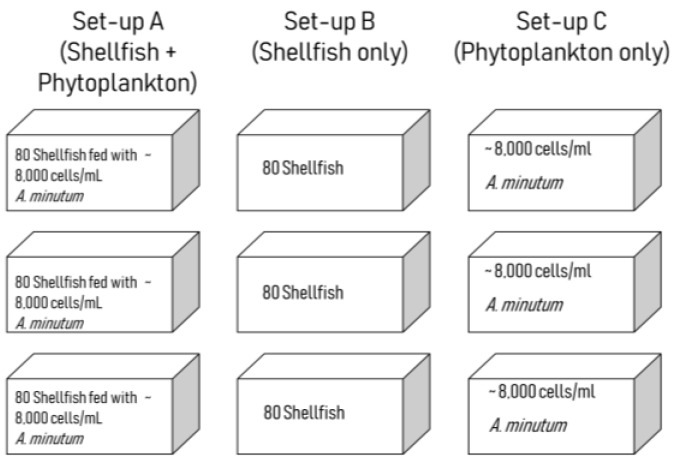
Schematic diagram of the experiment set-ups.

Three different treatments were implemented in 80 L aquarium tanks: Tank type A contained mussels with *A. minutum* (phytoplankton + shellfish), tank type B contained mussels only (shellfish) and tank type C contained *A. minutum* only (phytoplankton) (Figure 7). Three replicate tanks were set up per treatment resulting in a total of nine 80 L tanks. Eighty mussels were placed into each of the first six tanks (tank set-ups A and B). In the first treatment (A) shellfish were fed with toxic *A. minutum* using concentrations of approximately 8000 cells/mL. Feeding was only done at the start of the experiment with the exposure to toxic cells for two days only. After the 48th hour sampling, toxic cells were removed and the tank water replaced with filtered seawater. The second treatment (B) served as a negative control and contained 80 individuals of shellfish per replicate without feeding of any toxic algae. The last treatment (C) served as a positive control containing *A. minutum* in concentrations of approximately 8000 cells/mL, without the presence of any shellfish. All tanks had UV-filtered seawater with constant aeration. Sampling was performed every six hours for 24 h and daily thereafter up to seven days. Five individual mussels from each shellfish-containing tank were sampled at each sampling period. These mussels were then divided into three parts for toxin analysis: gills + mantle + gonads, foot + adductor muscle; and, lastly the digestive gland or gut. Individuals were dissected over ice to minimise temperature effects on toxin changes. Shellfish meat samples were then stored in a freezer until required for toxin analysis. All parts of the five individuals were pooled together before toxin analysis.

### 5.4. Toxin Analysis and Cell Counts

Toxin extraction of shellfish meat was conducted with the use of a ratio of 1:1 shellfish meat to 1 mL of 0.1 M hydrochloric acid [32]. Acid was added to tissue samples, vortex mixed then placed in a boiling water bath for five minutes. After boiling, the extract pH was adjusted to 3, centrifuged and supernatants filtered through a 0.2 µm syringe filter. Solid phase extraction (SPE) was used to clean-up the extracts as per Lawrence and Menard (1991) with modifications. A total of 250 µL of derivatizing agent (0.03 M HIO_4_, 0.3 M NH_4_HCO_3_, 0.3 M Na_3_PO_4_) with a ratio of 1:1:1, and 50 µL from the shellfish samples were incubated for 3 min [32]. CH_3_COOH (5.0 µL) was added prior to injection of a 50 µL aliquot of the reaction mix into the HPLC (LC-10A Shimadzu HPLC with RF-10AXL fluorescence detector). Separation used an Inertsil ODS-3V C18 (4.6 × 150 mm) column at 0.90 mL min^−1^ flow with binary gradient solvent system of 0.1 M ammonium formate (pH 7.0) for solvent A and HPLC-grade acetonitrile for solvent B. The elution gradient consisted of: 1% solvent B for 2 min, 1–5% solvent B for 3 min; 5% solvent B for 4 min; 5–6% solvent B for 1 min; 6–10% solvent B for 10 min.

Detection of PST analogues was conducted through comparison of oxidation product chromatographic peaks against those generated from working calibration standards prepared from Certified Reference Materials (CRMs) of STX, neo-STX, dcSTX, GTX1,4; and GTX2,3. CRMs were obtained from the Institute of Biotoxin Metrology, National Research Council, Canada. Chromatography and FLD was conducted according to AOAC 2005.06 [33].

Water (100 mL) was collected from the centre of each tank during every sampling period and used for toxin analysis. An additional 3 mL was collected near the surface, mid-depth, and bottom part of the tanks and used for phytoplankton counting to determine algal cell density through time. Cell counts were used to determine the clearance rate over time of mussel individuals.

Uptake and depuration rates were calculated by assessing the total toxicity accumulated over time by adding total toxin from each compartment within the shellfish tissues. The following equation was used for this computation

Uptake Rate/Depuration Rate = (TTCN − TTCN-1)/h(1)

where: TTCN = Total toxicity Concentration at time N.

Assimilation of toxin was measured by assessing the total toxicity present in the gut and other shellfish parts over time.

### 5.5. Data Analysis

The resulting data did not conform to assumptions of homogeneity and normality despite various transformations, thus non-parametric tests were used for statistical analyses. A multiple *t*-test was used to determine significant differences at each time point for overall toxicity of water, cell densities, in the phytoplankton only and shellfish fed with phytoplankton setups, and GTX values at different parts and in the water. A Kruskal–Wallis test was used to determine significant difference in overall toxicity across shellfish parts.

This paper is derived from the corresponding author’s master’s thesis [34]. 

## Figures and Tables

**Table 1:** Overall toxin concentrations in *P. viridis* for each PST analogue through time (mean ± S.E. in µg STX eq./100 g).

Time (h)	NeoSTX	STX	dcSTX	GTX 1,4	GTX 2,3
0	n.d.	n.d.	n.d.	0 ± 0.14	0 ± 0.04
6	n.d.	n.d.	n.d.	12.15 ± 3.05	9.71 ± 7.82
12	n.d.	0.60 ± 0.68	n.d.	22.14 ± 3.90	20.55 ± 17.57
18	n.d.	15.82 ± 14.45	0.10 ± 0.02	50.98 ± 31.95	36.30 ± 13.30
24	n.d.	0.78 ± 0.13	0.02 ± 0.03	26.37 ± 23.53	20.07 ± 28.44
48	n.d.	0.91 ± 0.66	0.01 ± 0.02	77.13 ± 70.38	164.9 ± 120.4
72	n.d.	0.50 ± 0.15	0.02 ± 0.02	8.05 ± 15.54	73.76 ± 44.09
96	n.d.	4.81 ± 1.81	n.d.	59.86 ± 54.36	302.1 ± 155.7
120	n.d.	2.82 ± 1.74	n.d.	183.6 ± 146.6	108.4 ± 62.92
144	n.d.	4.27 ± 2.47	n.d.	105.9 ± 61.12	195.5 ± 112.8

n.d.: not detectable

## Supplementary Materials

The following are available online at https://www.mdpi.com/2072-6651/11/8/468/s1, Table S1: Total toxicity in shellfish samples. Table S2: Toxicity of water in phytoplankton+shellfish set-up and phytoplankton only. Table S3: Toxin analogues of *A. minutum* and *P. viridis*. Figure S1: Image showing the parts of *Perna viridis* that were used for the study (Digestive Gland or Gut; Gills and Mantle; Adductor Muscle and Foot). Figure S2: Representative chromatograms from the HPLC for the Muscle (**A**), Gut (**B** and **C**), and Mantle (**D**). Standards for the toxins are overlaid on the diagrams and their peaks labeled.
