# Supplementary material for: Paralytic Shellfish Toxin Uptake, Assimilation, Depuration, and Transformation in the Southeast Asian Green-Lipped Mussel (Perna viridis)
Source: Toxins (Basel). 2019 Aug 9;11(8):468. doi: 10.3390/toxins11080468 (PMC6723038; doi:10.3390/toxins11080468)
Supplement: Supplementary file 1 [file toxins-11-00468-s001.pdf]

Supplementary Materials: Paralytic Shellfish Toxin Uptake, Assimilation, Depuration, and Transformation in the Southeast Asian Green-Lipped Mussel (*Perna viridis*)

John Kristoffer Andres, Aletta Yniguez, Jennifer Mary Maister, Andrew Turner, Dave Eldon Olano, Jenelyn Mendoza, Lilibeth Salvador-Reyes and Rhodora Azanza

Table S1. Total toxicity in shellfish samples.

	Total Toxicity (μg STX eq./100 g Shellfish Meat)					
Time (h)	Shellfish only			Phytoplankton + Shellfish		
0	1.198974	1.200621	1.421385	0	0	0
6	3.485035	1.468735	1.259965	9.861461	26.11228	29.6203
12	2.895207	2.05051	1.406038	66.9624	34.66052	28.24507
18	0.336124	0.171338	0.272465	88.31233	139.442	81.87709
24	0.460453	1.014181	5.607629	106.9203	18.58581	16.23498
48	13.29285	16.00297	14.57988	380.495	60.50999	287.7276
72	1.077198	15.98032	1.800508	136.8163	18.43388	91.75012
96	2.889174	0.617146	1.287261	406.0978	184.4196	509.6915
120	1.728666	0	1.391456	199.1898		390.4394
144	0.151518	0	0	305.5924		

Table S2. Toxicity of water in phytoplankton+shellfish set-up and phytoplankton only.

Time (h)	Total Toxicity (μg STX eq./100 mL)					
	Phytoplankton+Shellfish			Phytoplankton Only		
	Tank 1	Tank 2	Tank 3	Tank 1	Tank 2	Tank 3
0	18.12335	16.97658	4.398044	7.510502	2.282434	31.65408
6	10.37859	31.60624	38.82112	11.46775	1.65898	21.85389
12	31.9517	37.23117	42.68215	9.796581	1.731867	24.29144
18	14.84243	22.77435	19.73763	12.33506	1.843153	24.34668
24	5.304374	67.4956	21.79512	15.67713	2.191028	26.33178
48	5.056712	150.6173	42.25879	17.32389	2.441487	9.96633
72	61.39095	16.76608	9.421986	7.862049	8.047058	111.2079
96	3.861172	49.45741	12.14658	7.7709	2.000769	13.18208
120	3.840892		13.08046	10.62719		9.778759
144	9.05759			4.636389		

Table S3. Toxin analogues of *A. minutum* and *P. viridis.*

A.minutum Toxicity (µg STX eq./100 mL)					
**Time (h)**	**NSTX**	**GTX1,4**	**STX**	**dcSTX**	**GTX2,3**
0	0 ± 0	0.1146 ± 0.1519	0.0009 ± 0.0008	0.0001 ± 0.0001	0.0226 ± 0.0190
6	0 ± 0	0.0311 ± 0.0275	0.0018 ± 0.0019	0.0002 ± 0.0002	0.0835 ± 0.0716
12	0.0002 ± 0.0004	0.0461 ± 0.0401	0.0021 ± 0.0024	0.0006 ± 0.0006	0.0705 ± 0.0720
18	0.0003 ± 0.0004	0.0530 ± 0.0420	0.0020 ± 0.0022	0.0005 ± 0.0006	0.0726 ± 0.0689
24	0.00003 ± 0.000006	0.0650 ± 0.0604	0.0021 ± 0.0024	0 ± 0	0.0801 ± 0.0634
48	0.0052 ± 0.0055	0.0596 ± 0.0595	0.0018 ± 0.0020	0.0007 ± 0.0007	0.0318 ± 0.0277
72	0.0028 ± 0.0037	0.3716 ± 0.5651	0.0028 ± 0.0034	0.0005 ± 0.0005	0.0460 ± 0.0340
96	0.01887 ± 0.01587	0.0422 ± 0.0232	0.0016 ± 0.0016	0.0003 ± 0.0005	0.0353 ± 0.0246
120	0.0033 ± 0.0058	0.0304 ± 0.0191	0.0013 ± 0.0016	0.0001 ± 0.0001	0.0414 ± 0.0357
144	0 ± 0	0.0602 ± 0.0376	0.0015 ± 0.0026	0 ± 0	0.0223 ± 0.0343
P. viridis **Toxicity (µg STX eq./100 g Shellfish Meat)**					
**Time (h)**	**NSTX**	**GTX1,4**	**STX**	**dcSTX**	**GTX2,3**
0	0 ± 0	0 ± 0	0 ± 0	0.0029 ± 0.0050	0 ± 0
6	0 ± 0	12.1537 ± 3.0530	0 ± 0	0 ± 0	9.7110 ± 7.8191
12	0 ± 0	22.1407 ± 3.8972	0.6009 ± 0.6817	0 ± 0	20.5477 ± 17.5743
18	0 ± 0	50.9812 ± 31.9450	15.8293 ± 14.4543	0.0973 ± 0.0245	36.3027 ± 13.2971
24	0 ± 0	26.3744 ± 25.5330	0.7769 ± 0.1337	0.0188 ± 0.0326	20.0770 ± 28.4400
48	0 ± 0	77.1270 ± 70.3750	0.9102 ± 0.6568	0.0103 ± 0.0178	164.8633 ± 120.4447
72	0 ± 0	8.0505 ± 15.5405	0.5032 ± 0.1490	0.0221 ± 0.0196	73.7576 ± 44.0916
96	0 ± 0	59.8586 ± 54.3630	4.8085 ± 1.8072	0 ± 0	302.1003 ± 155.6853
120	0 ± 0	183.6167 ± 1443.1451	2.8151 ± 0.8863	0 ± 0	108.4402 ± 8.8787
144	0 ± 0	105.8658	4.2700	0	97.7283

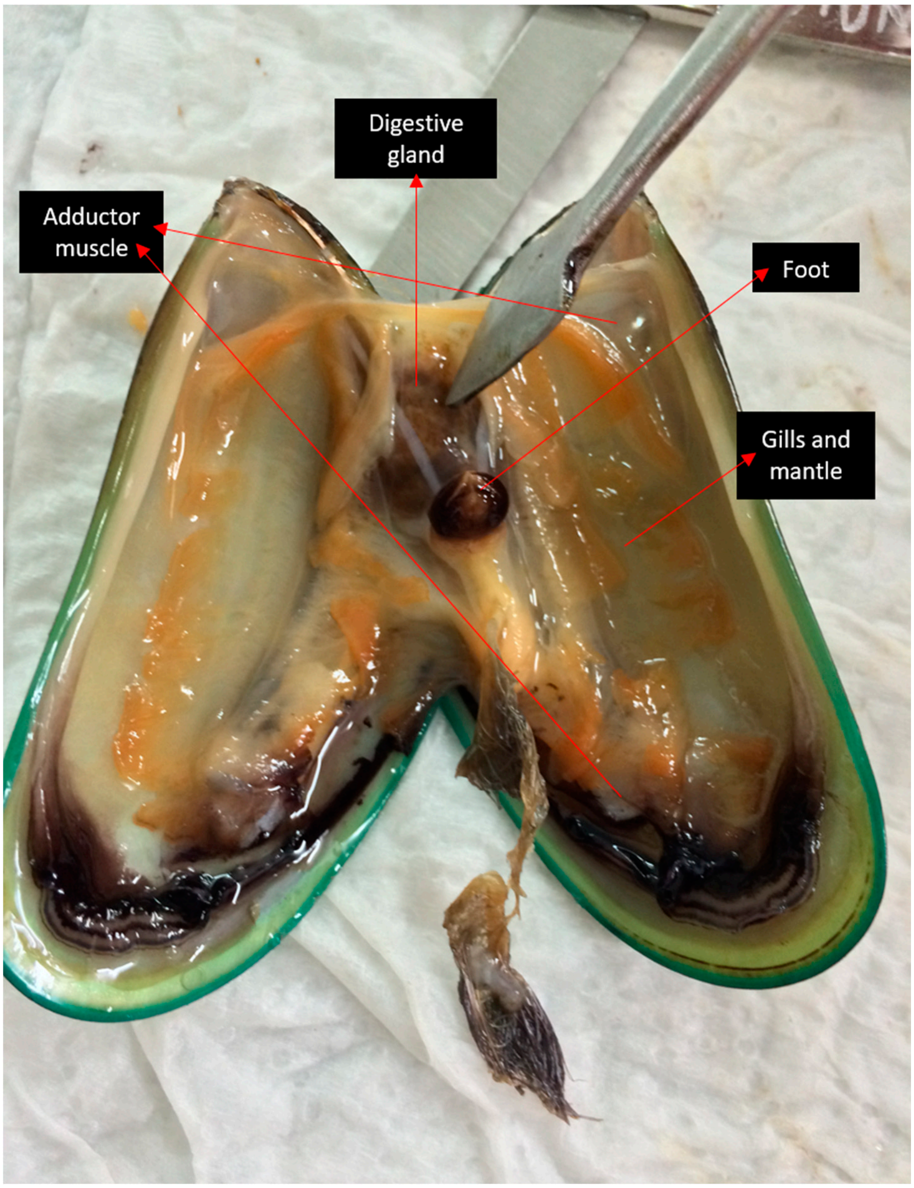

Figure S1. Image showing the parts of *Perna viridis* that were used for the study (Digestive Gland or Gut; Gills and Mantle; Adductor Muscle and Foot).

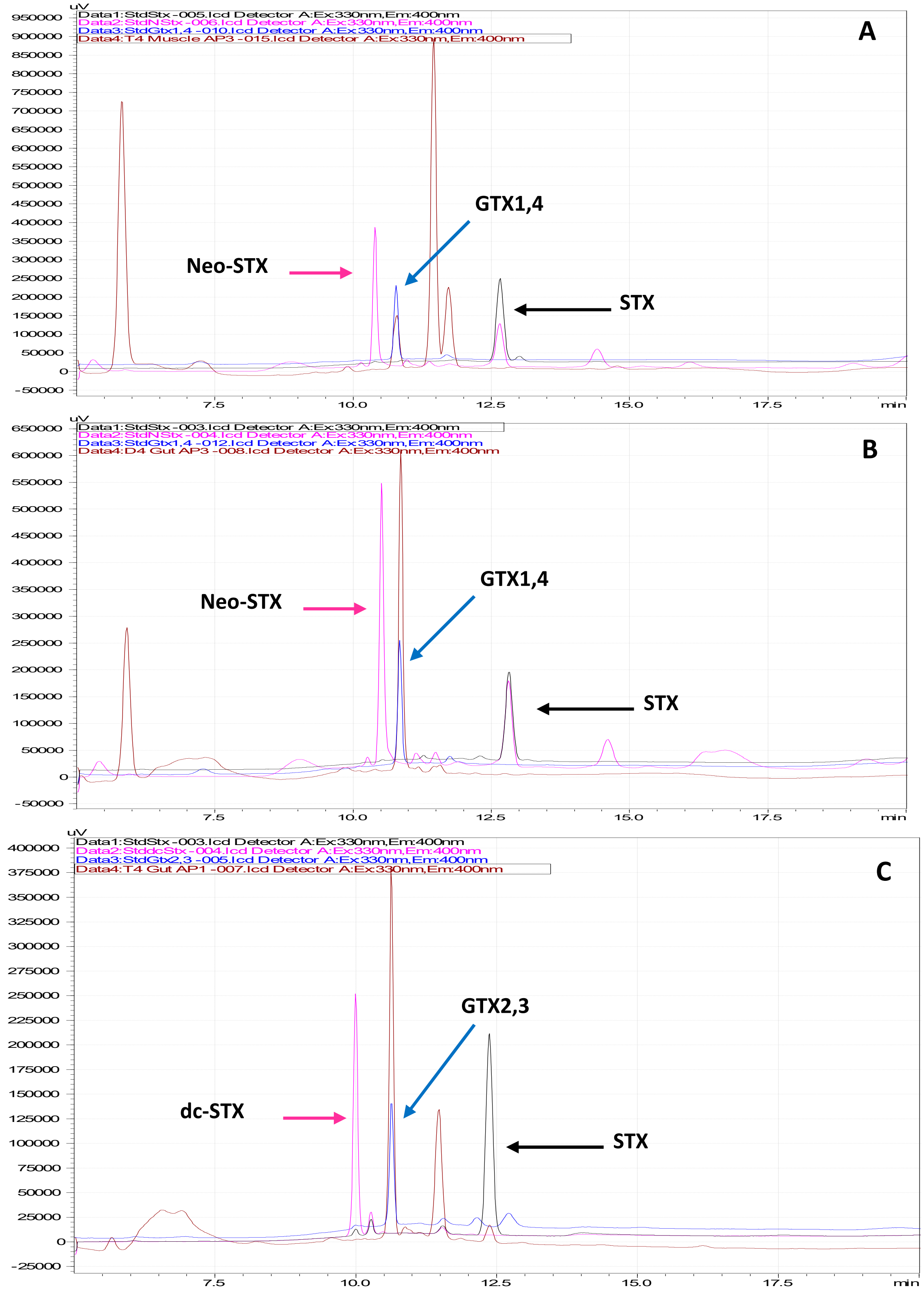
A
uV
Data1:StdStx-005.lcd Detector A:Ex330nm,Em:400nm
Data2:StdNStx-006.lcd Detector A:Ex330nm,Em:400nm
Data3:StdGtx1,4-010.lcd Detector A:Ex330nm,Em:400nm
Data4:T4 Muscle AP3-015.lcd Detector A:Ex330nm,Em:400nm
GTX1,4
Neo-STX
STX
min
B
uV
Data1:StdStx-003.lcd Detector A:Ex330nm,Em:400nm
Data2:StdNStx-004.lcd Detector A:Ex330nm,Em:400nm
Data3:StdGtx1,4-012.lcd Detector A:Ex330nm,Em:400nm
Data4:D4 Gut AP3-008.lcd Detector A:Ex330nm,Em:400nm
GTX1,4
Neo-STX
STX
min
C
uV
Data1:StdStx-003.lcd Detector A:Ex330nm,Em:400nm
Data2:StddcStx-004.lcd Detector A:Ex330nm,Em:400nm
Data3:StdGtx2,3-005.lcd Detector A:Ex330nm,Em:400nm
Data4:T4 Gut AP1-007.lcd Detector A:Ex330nm,Em:400nm
GTX2,3
dc-STX
STX
min

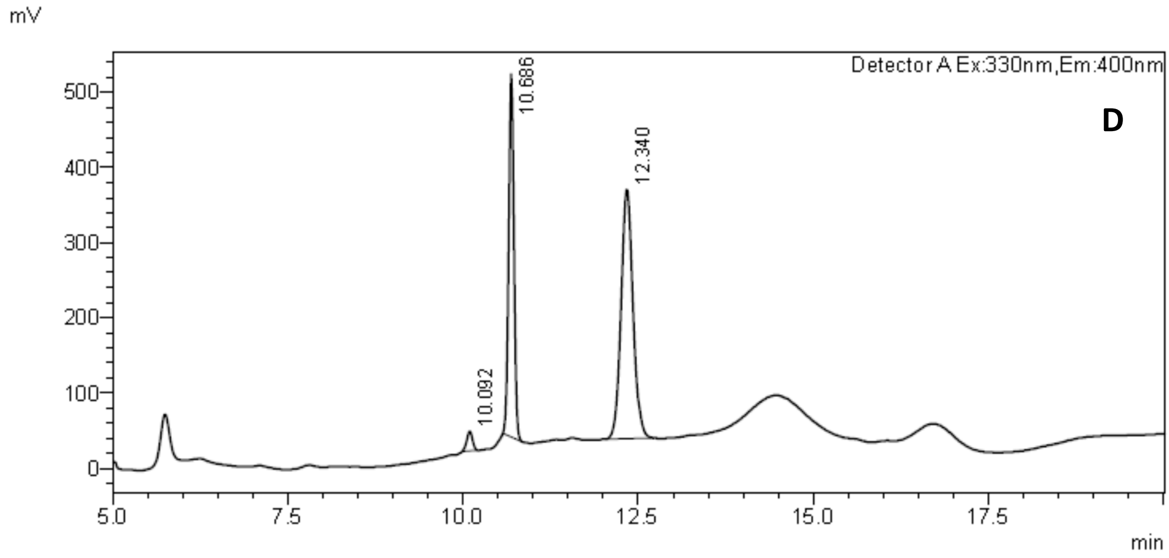

Figure S2. Representative chromatograms from the HPLC for the Muscle (**A**), Gut (**B** and **C**), and Mantle (**D**). Standards for the toxins are overlaid on the diagrams and their peaks labeled.
